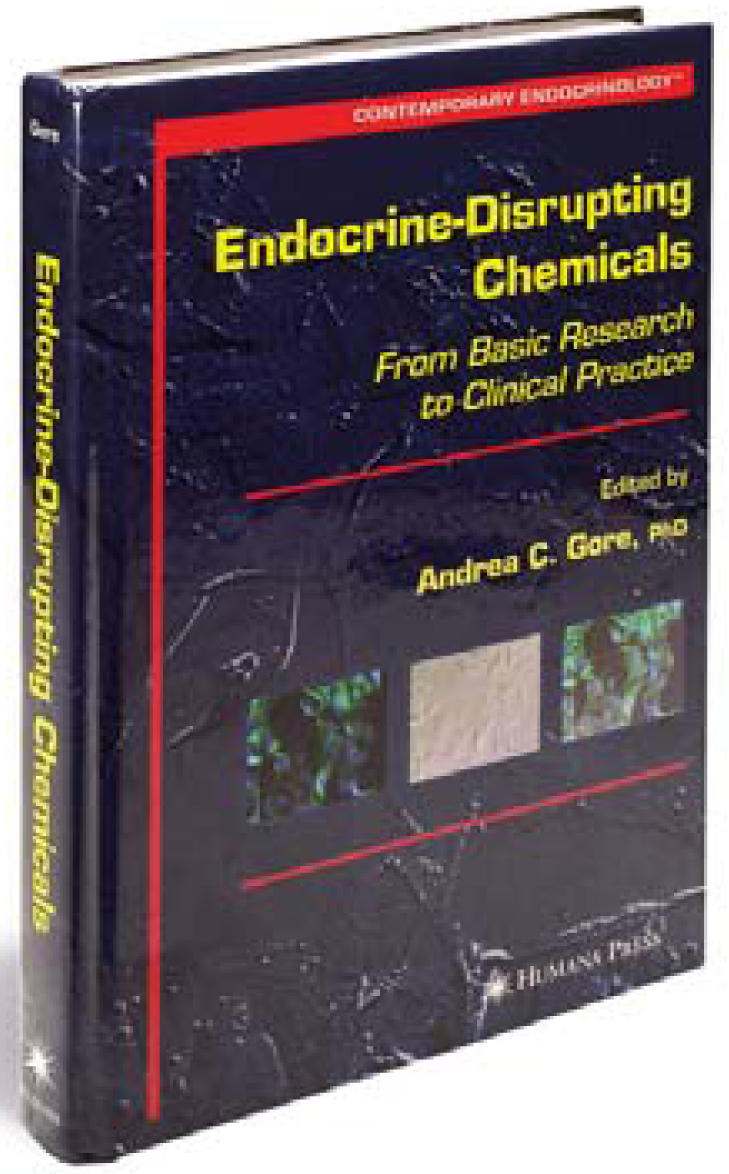# Endocrine-Disrupting Chemicals: From Basic Research to Clinical Practice

**Published:** 2008-04

**Authors:** Sylvia Hewitt

**Affiliations:** Sylvia Hewitt is a biologist with the Receptor Biology Group at the National Institute for Environmental Health Sciences in Research Triangle Park, North Carolina, since 1987. She is especially interested in mechanism of estrogenic moieties

*Endocrine-Disrupting Chemicals: From Basic Research to Clinical Practice*, is an aptly named volume comprising chapters that detail numerous aspects of endocrine disruptors from the perspectives of scientists, epidemiologists, and medical practitioners. Each group has its own scope and focus, but all are ultimately intertwined by a common goal of understanding what is necessary to ensure optimal human health.

The book, edited by Andrea Gore, is divided into three sections, the first focusing on the basic biology, the second detailing evidence for effects of endocrine disruptors on human health, and the third focusing on public policy, remediation, and interventions. The topics are not covered comprehensively, but the chapters might serve as a basic introduction to readers who need only a rudimentary understanding of the area, and the bibliography provides resources for those who wish to understand the topics in more depth.

The first part of the book includes seven chapters that introduce some of the diverse experimental models and approaches that illustrate potential endocrine-disrupting activities. These include developmental rodent models that focus on observed malformations and pathologies in the mammary gland, reproductive tracts, external genitalia, and the brain and the resulting disruptions in function of these tissues. A description of *in vitro* models for screening potential endocrine disruptors is also included. A very brief introduction to epigenetic effects is presented, but is a bit terse considering the current intense focus in this area of science. Various classes of potential disruptors are also laid out in this section. There is some overlap among the chapters, but readers can get the gist of current approaches and challenges.

The second part focuses on evidence for exposures that lead to effects on endocrine function in humans. One chapter briefly describes novel thyroid signaling mechanisms and introduces reported effects due to aromatic hydrocarbons, but rather briefly. Another chapter discusses observed effects of endocrine-disruptor exposures during different critical windows on fertility and reproductive functions in women. This includes collected evidence describing possible effects on menstrual cycles, development of endometriosis and polycystic ovary syndrome, menopause, as well as fertility and reproductive outcomes. Another chapter describes evidence for effects of endocrine disruptors on male reproductive health. This chapter compiles data regarding actual measurements of chemicals in body fluids and evaluation of end points such as semen quality, chryptorchidism, or hypospadias. The final chapter in this section discusses the appropriateness of various biomonitorting approaches in evaluation of exposures and biological effects.

The final part of the book addresses government policies, patient education, and ways to address patient questions, intervene in a community, and examine approaches to address potential threats from endocrine disruptors. This section focuses on U.S. issues, without regard to policies in other countries or continents that at the very least may be instructive to the readers. The description of a community-based participatory research model is fascinating and also suggests a successful approach to apply and remediate many of the issues laid out in the earlier sections of the book. The final chapter—“What Can We Do About Endocrine-Disrupting Chemicals?”—describes approaches used to tackle such questions as What should we consider to be an endocrine disruptor? How much exposure is acceptable? The chapter outlines current policies that “predict and permit,” which assume that there is a “safe” level of exposure, or policies that “identify and restrict,” an impossible and daunting task. The proposed approach is “green chemistry, clean production and precaution,” which involves engineering as little waste and pollution into production of safer compounds.

The issues addressed in this book—that endocrine disruptors threaten the critical reproductive processes and thus the future survival of humans and other species—are not likely to be resolved, but the cross-disciplinary descriptions included are important because they expand readers’ knowledge of the issues involved.

## Figures and Tables

**Figure:**